# Follicular thyroid carcinoma but not adenoma recruits tumor-associated macrophages by releasing CCL15

**DOI:** 10.1186/s12885-016-2114-7

**Published:** 2016-02-15

**Authors:** Feng-Jiao Huang, Xiao-Yi Zhou, Lei Ye, Xiao-Chun Fei, Shu Wang, Weiqing Wang, Guang Ning

**Affiliations:** Shanghai Key Laboratoryfor Endocrine Tumors, Shanghai Clinical Center for Endocrine and Metabolic Diseases, Shanghai Institute of Endocrine and Metabolic Diseases and Shanghai E-institute for Endocrinology, Ruijin Hospital, School of Medicine, Shanghai Jiao Tong University, 197 Ruijin 2nd Road, Shanghai, 200025 P.R. China; Department of Pathology, Ruijin Hospital, Shanghai Jiao Tong University, School of Medicine, 197 Ruijin 2nd Road, Shanghai, 200025 P.R. China; Laboratory for Endocrine & Metabolic Diseases of Institute of Health Science, Shanghai Jiao Tong University School of Medicine and Shanghai Institutes for Biological Sciences, Chinese Academy of Sciences, 227 South Chongqing Road, Shanghai, 200025 P.R. China

**Keywords:** Follicular thyroid carcinoma, Follicular adenoma, Tumor-associated macrophages, CCL15

## Abstract

**Background:**

The differential diagnosis of follicular thyroid carcinoma (FTC) and follicular adenoma (FA) before surgery is a clinical challenge. Many efforts have been made but most focusing on tumor cells, while the roles of tumor associated macrophages (TAMs) remained unclear in FTC. Here we analyzed the differences between TAMs in FTC and those in FA.

**Methods:**

We first analyzed the density of TAMs by CD68 immunostaining in 59 histologically confirmed FTCs and 47 FAs. Cytokines produced by FTC and FA were profiled using antibody array, and validated by quantitative PCR. Chemotaxis of monocyte THP-1 was induced by condition medium of FTC cell lines (FTC133 and WRO82-1) with and without anti-CCL15 neutralizing antibody. Finally, we analyzed CCL15 protein level in FTC and FA by immunohistochemistry.

**Results:**

The average density of CD68^+^ cells was 9.5 ± 5.4/field in FTC, significantly higher than that in FA (4.9 ± 3.4/field, *p* < 0.001). Subsequently profiling showed that CCL15 was the most abundant chemokine in FTC compared with FA. *CCL15* mRNA in FTC was 51.4-folds of that in FA. CM of FTC cell lines induced THP-1 cell chemotaxis by 33 ~ 77 %, and anti-CCL15 neutralizing antibody reduced THP-1 cell migration in a dose-dependent manner. Moreover, we observed positive CCL15 immunostaining in 67.8 % of FTCs compared with 23.4 % of FAs.

**Conclusion:**

Our study suggested FTC might induce TAMs infiltration by producing CCL15. Measurement of TAMs and CCL15 in follicular thyroid lesions may be applied clinically to differentiate FTC from FA pre-operation.

**Electronic supplementary material:**

The online version of this article (doi:10.1186/s12885-016-2114-7) contains supplementary material, which is available to authorized users.

## Background

Fine-needle aspiration (FNA) biopsy has been recommended as the most accurate and cost-effective method for evaluating thyroid nodules [1, 2]. However, its accuracy has been largely compromised for follicular lesions, namely follicular thyroid carcinoma (FTC) and follicular adenoma (FA). The key feature of FTC to distinguish it from FA is capsular or vesicular invasion, which cannot be detected by either ultrasound or FNA cytology [3, 4]. Biomarkers for distinguishing FTC from FA before surgery are much needed.

Tumor associated macrophages (TAMs) play an important role in tumorigenesis and progression. TAMs generate an inflammatory environment to trigger or facilitate tumor initiation, promote tumor cell invasion and metastasis, stimulate angiogenesis and suppress antitumor immunity [5–7]. High density of TAMs was correlated with the poor prognosis of a wide range of tumors such as lung, hepatocellular, colorectal, breast, prostate, ovarian and thyroid cancers [8–13]. TAMs produced growth factors (e.g. VEGF, EGF, HGF and bFGF) and chemokines (e.g. CXCL12 and IL8) to mediate their oncogenesis function [7, 14]. On the other hand, cancer cells recruit TAMs by releasing colony stimulating factor (CSF1), granulocyte–monocyte (GM-CSF), transforming growth factor (TGF) or chemokines (e.g. CCL2) [15, 16]. These factors are potential candidate biomarkers for early diagnoses, prognosis evaluation or therapeutic targets of malignancy.

The first direct evidence of macrophages infiltration in thyroid cancers was found in 1994 [17]. Subsequent studies successively suggested positive association between increased TAMs density and thyroid cancer progression [18–20]. Mice study showed that CSF1/CSF1R mediated TAMs recruitment in papillary thyroid cancer (PTC) and targeting CSF1/CSF1R impaired BRAF-induced thyroid cancer progression [21]. Recently, we reported that TAM in PTC facilitated tumor metastasis through releasing CXCL8 and may target CXCR1/2 in tumor cells [22]. However, the roles of TAMs are still largely unknown in FTC. Noting that the capsular/vascular invasion featured FTC from FA, and TAMs promote tumor cell invasion, we proposed TAMs and their associated cytokines should also feature FTC and may be used as pre-operation biomarkers to distinguish FTC from FA.

In this study, we first investigated TAMs density in follicular lesions by using patient samples, and then explored cytokines responsible for TAMs recruitment and their roles in differentiating FTC from FA.

## Methods

### Patients

Paraffin embedded tissue specimens were obtained from 59 patients histologically confirmed as FTC and 47 patients with FA, and eight fresh-frozen tissue samples (four FTCs and four FAs) collected from surgical specimens, from 2010 to 2012 at Ruijin Hospital, Shanghai Jiaotong University, School of Medicine. Written informed consents were obtained from all patients. This study was approved by the board of medical ethics of Ruijin Hospital.

### Cell culture and reagents

FTC133 human cancer cell line was originally from Dr. Robert Gagel (University of Texas, M. D Anderson Cancer Center, Houston, Texas), and WRO82-1 cell line was get from Dr. Zhimin Liu (Chongqing Medical University). Both of them have been genetically fingerprinted by either single-nucleotide polymorphism comparative genome hybridization (SNP-CGH) and verified to be unique [23]. THP-1 cell line was obtained from the American Type Culture Collection (ATCC, Manassas, VA). Both WRO82-1 and THP-1 were cultured in RPMI 1640 supplemented with 10 % heated fetal bovine serum and 1 % LG and 1 % PS. FTC133 cell line was cultured in DMEM/modified HAM-F12 medium. All medium were bought from Gibico (Rockville, MD). The passage numbers of all cell lines were within 30 passages.

Recombinant human CCL15 was purchased from Peprotech (rocky hill, USA). Affinity-purified anti-human CCL15 antibody and normal goat IgG were obtained from R&D Systems (Minneapolis, USA).

### Tissue microarrays and immunohistochemistry

Tissue microarrays (TMAs) were constructed using 2 mm cores from formalin-fixed, paraffin-embedded tissue blocks. Immunostaining of CD68 (1:150; Dako, Glostrup, Denmark), CD163 (1:75; Vector Laboratories, Burlingame, CA), CD206 (1:5000; Abcam, Cambrige, UK) and CCL15 (1:250; Novus Biologicals, USA) were performed on section of 4 μm thickness as previously reported [13]. Briefly, the first antibody was incubated at 4 °C overnight before the secondary antibody conjugated with horseradish peroxidase was added. DAB (3,3-diami-nopdbenzidine) substrate was introduced and hematoxylin was used for counterstaining. The stained sections were microscaned by nanozoomer2.0-RS (Hamamatsu) to get the photo of entire sample. A single pathologist, who was blinded to the histological assessments of each case, counted the number of CD68/CD163/CD206 positive cells. For tissue microarrays sections, CD68, CD163 or CD206 positive cells were counted in five independent fields under 400 magnifications (represent 0.06 mm^2^). For whole tissue sections, CD68^+^ cells in ten independent fields under 400 magnifications (represent 0.06 mm^2^) were counted. To avoid any overestimation of the number of TAMs which could have been due to extended cytoplasmic ramifications, we counted only cells with a visible nucleus. The CCL15 staining level was evaluated by an expert pathologist and was classified as negative (CCL15-) and positive (CCL15+, ++).

### Cytokine antibody array

Surgically resected tissue samples were obtained from four FTC and two FA patients. RayBio Human Cytokine Antibody Array G Series was purchased from RayBiotech (Norcross, GA). According to the manufacturer’s instruction, array membranes were incubated for 30 min in blocking buffer and afterwards in tissue lysates with 120 μg total protein overnight at 4 °C. The membranes were washed and a diluted cocktail of biotin-conjugated antibodies was added for 2 h. Membranes were washed again and the sandwiched antigens were detected by incubation with a fluorescent dye-conjugated streptavidin solution for 2 h. Signals were captured by laser scanner Axon GenenPix using Cy3 and analyzed by RayBio Antibody Array Analysis Tool.

### RNA extraction and qRT-PCR

Total RNA was isolated from FTC or FA tissue samples, and THP-1 cells under different treatments using TRIZOL reagent (Invitrogen) and 1 mg total RNA was converted into first-strand cDNA with the First Strand cDNA Synthesis Kit (Promega, USA) according to the manufacturer’s instructions. qRT-PCR was performed in Light Cycler480 instrument (Roche Diagnostics, Switzerland), using the SYBR Premix Ex TaqTM (TaKaRa, Japan). The expression data were analyzed using △△Ct and ACTB was used as internal control. Primers used were list in Additional file 1: Table S5.

### Chemotaxis assays

Chemotaxis assays were performed using 8 μm pore size cell culture inserts within 24-well plates (Corning, USA) as previously described [22]. FTC133 or WRO82-1 in exponential phase were cultured with FBS free medium (plus 0.2 % BSA, Genebase Gene-Tech, China) for 24 h and then the medium was harvested as condition medium (CM). THP-1 cells (5 × 10^5^/ml) were seeded into the inserts pre-coated with collagen from rat tail (Sigma, USA), and CM/ mock medium /CCL15 neutralizing antibody was added in the lower well. After 12 h of incubation, cells in the upper membrane surface were removed with cotton swabs. Cells on the lower membrane surface were fixed with methyl alcohol and stained with 0.1 % crystal violet (Sigma, USA). The cells of five randomly selected fields per well were counted using the Axiovert 25 microscope (Carl Zeiss, Germany) under 400 magnifications. Three replicate measurements were included in a single experiment.

### Statistical analysis

Statistical analyses were performed by SPSS Version 17.0 and Prism Version 5.0. Data was expressed as mean ± standard deviation(SD). Mann–Whitney test was used for continuous variables between groups, while Fisher’s exact test for counts between categorical variables. All *p* values were 2-tailed, and *p* < 0.05 was accepted as statistical significance.

## Results

### TAMs density was increased in FTC compared with FA

**Fig. 1:**
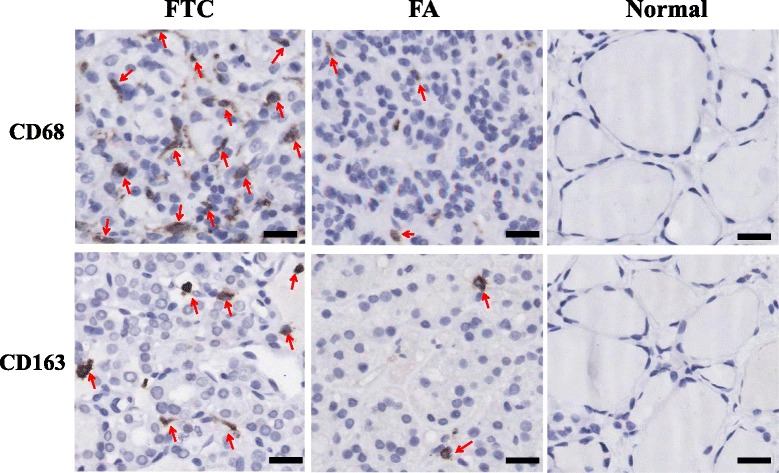
Densities of CD68+ and CD163+ cells in FTCs are significantly higher than those in FAs. Immunohistochemistry analysis of CD68 and CD163 in 158 tissue samples of FTC and FA patients. Normal: adjacent non-neoplastic tissues which exhibit normal pathological phenotypes. Arrows indicate the CD68+ or CD163+ macrophages. Bar = 20 μm

We previously found that TAMs increased PTC cancer progression through CXCL8 [13, 22]. To investigate whether TAM plays pathologic roles in FTC, we firstly quantified the amounts of TAMs in follicular thyroid lesions. The macrophage marker CD68 was immunostained in 158 tissue blocks obtained from 106 patients (including 59 FTCs and 47 FAs). We observed CD68 positive cells unevenly distributed in the lumen of the follicles or interspersed between the tumor cells, but rarely appeared in non- neoplastic thyroid tissues (Fig. 1). The average density of CD68^+^ cells was 9.5 ± 5.4/field in the FTCs, significantly higher than that in benign thyroid lesions (4.9 ± 3.4 for FA and 1.3 ± 1.2 for normal thyroid tissue, *p* = 1.3 × 10^−6^ /3.4 × 10^−19^, Mann–Whitney, Table 1 and Fig. 1). In order to test whether tissue microarray is representative of the entire lesion, we further immunostained CD68 in the tissue sections of 20 FTCs and 20 FAs. A total of ten fields under 400X (an area of 300 μm*200 μm) were counted for each sample. The result showed the same, as significantly higher CD68+ cells in FTC than in FA (7.7 ± 3.7/field versus3.7 ± 1.4/field, *p* = 7.3 × 10^−5^, while on TMAs 10.7 ± 5.3/field versus 4.2 ± 1.9/field, *p* = 6.8 × 10^−6^; Additional file 1: Table S1 and Additional file 2: Figure S1).

**Table 1:** Increased Density of CD68 Positive Macrophages in FTCs comparing to FAs

Histological Type	Case number	TAM counts (Mean /field)	*P* value
FTC	59	9.5 ± 5.4	
FA	47	4.9 ± 3.4	1.3 × 10^−6^
Normal Tissue	55	1.3 ± 1.2	3.4 × 10^−19^

We also compared the immunostaining of CD163 and CD206, two markers of M2 subgroup of TAMs, in FTCs and FAs. We found FTC harbored a significantly higher density of M2 TAMs compared with FA. The number of CD163^+^ cells was 6.3 ± 3.9/field in FTC versus 4.6 ± 4.3/field in FA (Mann–Whitney, *p* = 0.001, Fig. 1, Table 2); and CD206^+^ cells in FTC and FA were 4.3 ± 2.0/field and 3.0 ± 2.4/field respectively (Mann–Whitney, *p* = 0.020; Additional file 1: Table S2 and Additional file 2: Figure S2).

**Table 2:** Increased Density of CD163 Positive Macrophages in FTCs comparing to FAs

Histological Type	Case number	TAM counts (Mean /field)	*P* value
FTC	59	6.3 ± 3.9	
FA	46	4.6 ± 4.3	0.001

Furthermore, we investigated the relationship between CD68^+^ macrophages density and the clinicopathological features of FTC patients. We found no significant correlation between TAM density and TNM stages, capsular invasion and vascular invasion (Additional file 1: Table S3).

### CCL15 was the most abundant chemokine in FTC comparing with FA

**Fig. 2:**
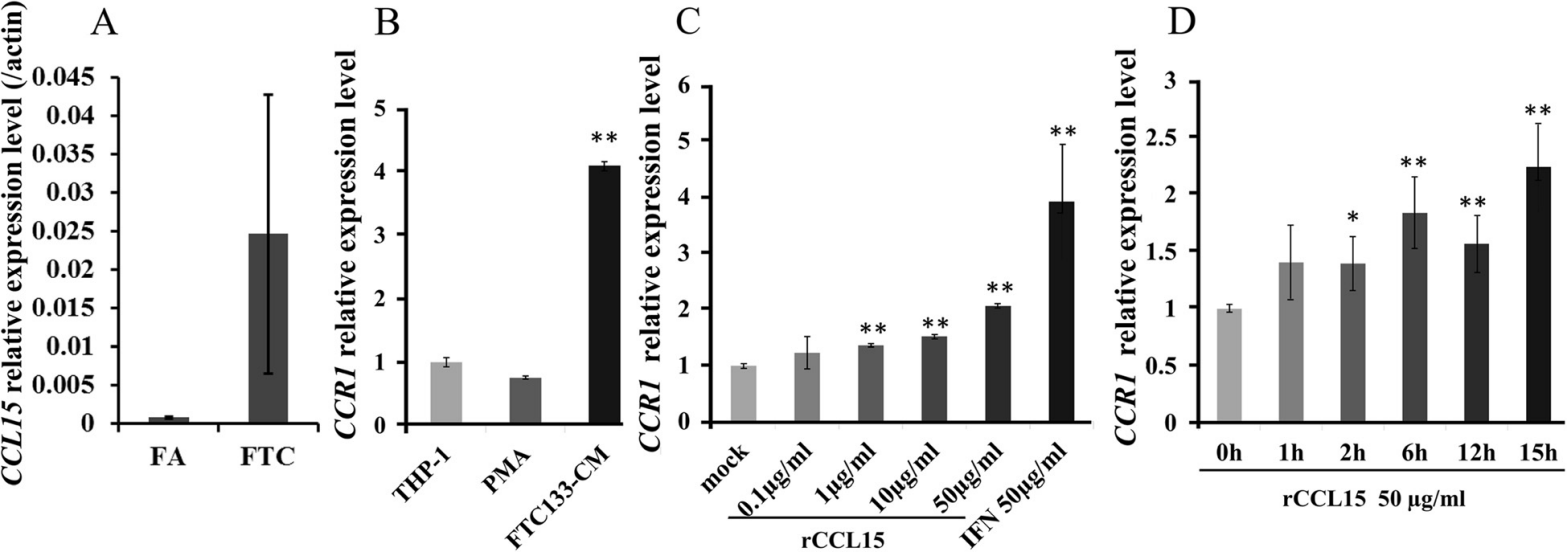
*CCL15* is highly expressed in FTCs and *CCR1* is induced by FTC133-CM. **a** qRT-PCR analysis of the CCL15 expression level in fresh tissues of FTC and FA tissues. Relative expression was normalized by the expression of ACTB. Data was presented as mean ± SE (*n* = 4). **b**, **c**, **d** qRT-PCR analysis of the CCR1 expression level in Thp-1 cell line. Thp-1 cells were treated with 30 ng/ml PMA or conditional media of FTC133 (FTC133-CM) for 24 h (**b**); Thp-1 cells were treated with increasing doses of rCCL15 for 24 h, and 50 μM IFN was used as a positive control (**c**); Thp-1 cells were treated with 50 μg/ml rCCL15 for increasing time periods (**d**). Relative expression was normalized by the expression of CCR1 in Thp-1 under mock treatment. ACTB was used as internal control and data were presented as mean ± SD (*n* = 3). Experiments were repeated three times, with consistent results

To discover the specific chemokines that recruit TAM accumulation in FTC, the abundance of 80 cytokines, chemokines and growth factors were examined by cytokine antibody array in protein lysis from fresh FTC and FA tissues. We found that CCL15 was the most abundant chemokine in FTC when compared with FA. We confirmed this finding by qRT-PCR, which showed *CCL15* mRNA in FTC was 51.4-folds of that in FA (Fig. 2a). Moreover, the expression of *CCR1* (CCL15 receptor) in monocytes cell line THP-1 can be induced by condition media of FTC133 cell line (FTC133-CM) (Fig. 2b) and by recombinant CCL15 (rCCL15) in dose-dependent and time-dependent manners (Fig. 2c, d).

**Fig. 3:**
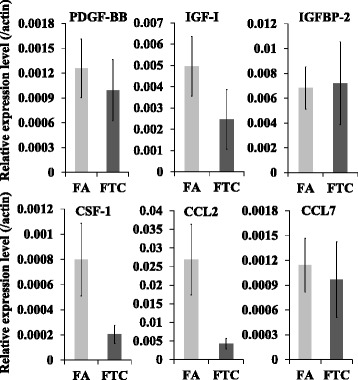
Expression of another 6 candidates in FA and FTC. mRNA levels of PDGF-BB, IGF-I, IGFBP-2, CSF-1, CCL2 and CCL7 were analyzed by qRT-PCR in fresh tissues of FTC or FA. Relative expression was calculated using the expression level of ACTB as internal control and data was presented as mean ± SE (*n* = 4)

We tested the mRNA levels of six more genes, including three with different abundance in antibody array between FTC and FA, two previously reported TAMs recruiters (*CCL2* and *CCL7*), and *CSF-1* which was reported as TAMs recruiters in thyroid cancers. However, none of them exhibited significantly higher expression in FTC than in FA (Fig. 3).

### FTC cancer cells recruited macrophages by secreting CCL15

**Fig. 4:**
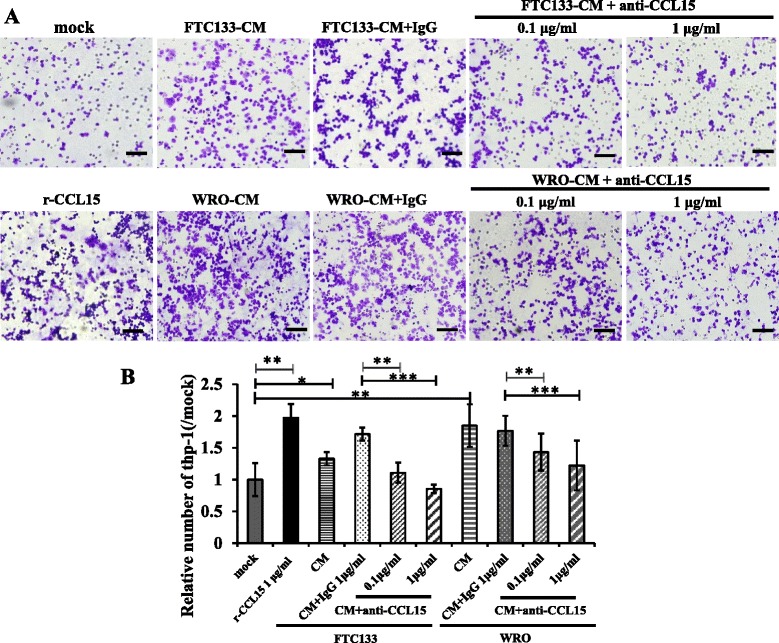
CCL15 mediates the recruitment of macrophages in FTC. **a** Transwell assay of Thp-1 cell line by different treatment for 12 h. Bar = 10 μm. **b** Thp-1 cell numbers were calculated in each field under 400 magnifications. Both rCCL15 and condition medium of FTC cancer cell lines (FTC133-CM and WRO-CM) enhanced the recruitment of Thp-1 (*p < 0.05, **p < 0.01, compared with serum-free medium). Anti-CCL15 neutralizing antibody abrogated the recruitment by FTC133-CM or WRO-CM. (**p < 0.01, ***p < 0.001, compared with condition medium plus isotype-matched IgG). Statistical analysis was performed using a two-tailed Student’s *t*-test and the data was presented as mean ± SD (*n* = 16). Three biological replications were performed, with consistent results

To confirm that CCL15 directly contributes to macrophage recruitment, we performed transwell assay of monocytes cell line THP-1 by using condition medium of FTC cell lines WRO82-1 and FTC133. Compared with mock medium, FTC133-CM and WRO82-1-CM induced THP-1 cell chemotaxis by 33 and 77 %, respectively (Fig. 4). Moreover, rCCL15 enhanced THP-1 cell migration by 97 %. When CM of FTC133 or WRO82-1was pretreated with anti-CCL15 neutralizing antibody, we observed reduced THP-1 cell migration in a dose-dependent manner (35.4 % at 0.1 μg/ml, and 50.2 % at 1 μg/ml for FTC133; 18.9 % at 0.1 μg/ml, and 30.8 % at 1 μg/ml for WRO82-1) (Fig. 4). Collectively, these data suggested that FTC tumor cell-derived CCL15 played an important role in recruiting macrophages in FTC.

### CCL15 was significantly highly expressed in FTC than in FA

**Fig. 5:**
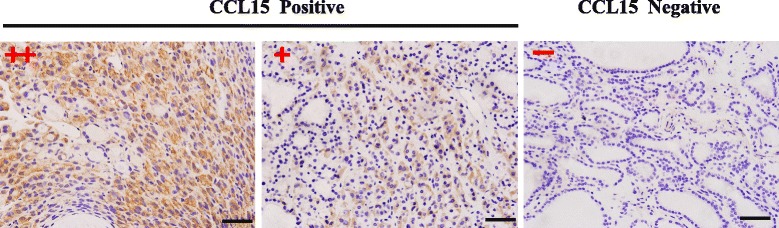
Immunostaining of CCL15 in thyroid follicular lesions. “+” and “-” represents the stage of staining level, which were estimated by pathologist, who was blind to the histological assessments of each case. Bar = 20 μm

To test whether CCL15 was differentially expressed in FTC and FA, we compared CCL15 immunostaining in FTC or FA tissue blocks (Fig. 5). We observed positive CCL15 immunostaining in 67.8 % of FTCs compared with 23.4 % of FAs. (Fisher’s exact test, *p* = 1.4 × 10^−5^; Table 3). Interestingly, tumors CCL15 positive expression also possessed high CD68^+^ macrophage density, (8.5 ± 5.3/field in CCL15 positive cases versus 6.4 ± 4.8/field in CCL15 negative cases, *p* = 0.01, Mann–Whitney test; Table 3). In consistent with the density of TAMs, no correlations between CCL15 expression level and clinicopathological features of FTC patients were observed (Additional file 1: Table S4).

**Table 3:** Correlation of CCL15 expression and CD68 positive macrophage in FTC and FA

Histological Type	CCL15 Positive Expression	CCL15 Negative Expression	*P* value
FTC	40(67.8 %)	19(32.2 %)	
FA	11(23.4 %)	36(76.6 %)	1.4 × 10^−5^
CD68	8.5 ± 5.3 (*n* = 51)	6.4 ± 4.8 (*n* = 55)	0.01

## Discussion

In this study we found FTC produced high level of CCL15 and recruited higher density of TAMs in FTC compared to FA. These important characters of FTC may have clinical significance in differentiating FTC from FA pre-operation.

The inflammatory microenvironment and macrophages infiltration have been demonstrated to be correlated with thyroid cancer progression and prognosis [20]. Our previous work also indicated that the density of TAMs was increased in advanced papillary thyroid cancers (PTC). However, the role of TAM in FTC remained unclear. In the current study, we firstly found TAMs was significantly accumulated in FTC comparing to its benign control. TAMs can produce chemokines to promote tumor invasion and metastasis [24]. As in PTC, we found TAMs density was correlated with lymph node metastasis and advanced tumor stage [13]. However, here we found no correlations between the density of TAMs and the clinicopathological features of FTC patients. The lack of correlation may result from limited sample size, especially those with advanced TNM stage. Alternatively, different from in PTC, TAMs might only function in the early phase of FTC tumorigenesis, independent with tumor progress events. The underlying mechanism is worth further studies.

Cancer cells recruit TAMs by producing specific chemokines, which may be applied clinically as biomarkers of malignancy. In our study, we confirmed CCL15 produced by FTC cell was responsible for TAMs recruitment in FTC in vitro. The CC motif chemokine ligand 15 (CCL15) is a potent chemoattractant for leukocytes and endothelial cells (ECs), and act through a receptor protein CCR1 [25, 26]. Human colon cancer cells produced CCL15 to recruit immature myeloid cells (iMCs) from bone marrow and then promoted tumor invasion in the invasion front [27, 28]. CCL15 level was associated with liver metastases in patients with colon cancer [29]. We also found significant high level of CCL15 in FTC comparing to FA. However, we failed to observe correlation between CCL15 expression and tumor stage. This is consistent with the lack of correlation between TAMs and FTC clinicopathological features. The influence of limited sample size was not excluded.

Preoperative diagnosis of FTC (especially minimally invasive FTC) has been a clinical challenge. In the last decade, specific molecular biomarkers have been proposed to improve preoperative diagnosis of FTC. These included immunostaining markers such as galectin-3 combined with CD44v6, p27, cytokeratin19 or HBME1 [3, 30–32]. Moreover, mRNA or miRNA expression profiles have identified a group of genes to distinguish FTC from FA [33–38]. Although these approaches improved our knowledge on FTC, all of them showed their limitations, such as limited operability or stability in clinical practice. All the previous efforts have focused on neoplasms themselves. The great heterogeneity harbored by individual tumors hampered the identification of a universal biomarker to differentiate FTC from FA. Currently, the golden diagnostic criteria for FTC are capsular invasion or angioinvasion, in which tumor microenvironment (TME) plays an important role. We proposed that FTC and FA might harbor different TME and the differences might have diagnostic values. Indeed, the current study indicated that TAMs, the most important cell component in TME, specifically accumulated in FTC but not in FA. More importantly, we identified that CCL15, which recruited TAMs in FTC, was significantly highly expressed in FTC. To our best knowledge, this is the first study trying to understand FTC and FA in the aspect of TME. However, the sensitivity and specificity of CCL15 as a marker to differentiate FTC from FA was not investigated because of sample limit. Future study should work on that.

## Conclusions

In this study, we found FTC recruited high density of TAMs by releasing CCL15, which may be applied clinically to differentiate FTC from FA pre-operation. The current work revealed the importance role of TAMs in FTC. Investigation in tumor microenvironment in addition to tumor cell may bring new insights into not only the pathogenesis but also early diagnosis of FTC.

## Additional files

Additional file 1: Table S1-S5. Comparison of CD68 immunostain in FTCs/FAs in tissue microarray and in the corresponding entire tissue sample sections. **Table S2.** Increased Density of CD206 Positive Macrophages in FTCs comparing to FAs. **Table S3.** Correlations of CD68 Positive TAMs Density with Clinicopathological Features of FTC Patients. **Table S4.** Correlations of CCL15 expression level with Clinicopathological Features of FTC Patients. **Table S5.** Sequence of primers used in this study. (DOCX 25.5 kb) Additional file 2: Figure S1 and S2. Immunostain of CD68 in entire tissue of FTC/FA samples. (A) An example of immunohistochemistry analysis of CD68 in whole tissue samples of FTC (left panel) and FA (right panel). Stars indicated blank areas were taked out for tissue microarrays constructing. CD68^+^ cells in ten 200 μm *300 μm areas (green-red marked) of every sample were counted. Bar = 1 mm. (B) Enlarged picture of one count area (one green-red area in A). Arrows indicate the CD68^+^ macrophages in FTC (left panel) or FA (right panel). Bar = 20 μm. **Figure S2.** Densities of CD206+ cells in FTC are significantly higher than those in FA. Immunohistochemistry analysis of CD206 in 55 cases of tissue samples from FTC and FA patients. Arrows indicate the CD206+ macrophages. Bar = 20 μm. (PDF 509 kb)

## Abbreviations

CCL15: CC motif chemokine ligand 15
FA: Follicular adenoma
FNA: Fine-needle aspiration
FTC: Follicular thyroid carcinoma
PTC: Papillary thyroid cancers
TAM: Tumor associated macrophages
TME: Tumor microenvironment
TNM: TNM Classification of Malignant Tumors
