# Supplementary material for: Follicular thyroid carcinoma but not adenoma recruits tumor-associated macrophages by releasing CCL15
Source: BMC Cancer. 2016 Feb 15;16:98. doi: 10.1186/s12885-016-2114-7 (PMC4753660; doi:10.1186/s12885-016-2114-7)
Supplement: Additional file 1: Table S1-S5. — Comparison of CD68 immunostain in FTCs/FAs in tissue microarray and in the corresponding entire tissue sample sections. Table S2. Increased Density of CD206 Positive Macrophages in FTCs comparing to FAs. Table S3. Correlations of CD68 Positive TAMs Density with Clinicopathological Features of FTC Patients. Table S4. Correlations of CCL15 expression level with Clinicopathological Features of FTC Patients. Table S5. Sequence of primers used in this study. (DOCX 25.5 kb) [file 12885_2016_2114_MOESM1_ESM.docx]

**Supplementary Data**

**Supplementary Table S1 Compare of CD68 immunostain in FTCs/FAs in tissue microarray and in the corresponding entire tissue sample sections**

|  | FTC | FA | *P* value |
| --- | --- | --- | --- |
| Case number | 20 | 20 |  |
| Microarray (Mean /field) | 10.7±5.3 | 4.2±1.9 | 6.8×10^-6^ |
| Whole Tissue (Mean /field) | 7.7±3.7 | 3.7±1.4 | 7.3×10^-5^ |

**Supplementary Table S2 Increased density of CD206 positive macrophages in FTC comparing to FA**

| Histological Type | Case number | TAM counts (Mean /field) | *P* value |
| --- | --- | --- | --- |
| FTC | 30 | 4.3±2.0 |  |
| FA | 25 | 3.0±2.4 | 0.020 |

**Supplementary Table S3 Correlations of CD68 positive TAMs density with clinicopathological features of FTC patients.**

|  | Patient (%) | CD68^+^counts | *P* value |
| --- | --- | --- | --- |
| Gender  Male  Female | 22(37.3%)  37(62.7%) | 8.36±4.41  10.17±5.82 | 0.35 |
| Age at diagnosis, year  <45  ≥45 | 27(45.8%)  32(54.2%) | 9.87±5.45  9.17±5.36 | 0.55 |
| T  T1/T2  T3/T4 | 46(83.6%)  9(16.4%) | 9.83±5.64  7.40±3.93 | 0.23 |
| N  No  Yes | 53(96.4%)  2(3.6%) | 9.63±5.60  6.80±0.00 | 0.75 |
| M  No  Yes | 54(98.2%)  1(1.8%) | 9.57±5.56  7 | 0.95 |
| STAGE  Ⅰ/Ⅱ  Ⅲ/Ⅳ | 48(87.3%)  7(12.7%) | 9.73±5.70  8.11±4.22 | 0.56 |
| Capsular invasion  Yes  No | 56(96.6%)  2(3.4%) | 9.50±5.51  9.50±0.42 | 0.73 |
| Vascular invasion  Yes  No | 23(39.7%)  35(60.3%) | 10.20±5.25  9.04±5.55 | 0.26 |

**Supplementary Table S4 Correlations of CCL15 Expression with Clinicopathological Features of FTC Patients.**

|  | No. | Negative Expression | Positive Expression | P value |
| --- | --- | --- | --- | --- |
| Gender |  |  |  |  |
| Male | 22 | 7 | 15 | 0.961 |
| Female | 37 | 12 | 25 |  |
| Age at diagnosis, year |  |  |  |  |
| <45 | 27 | 8 | 19 | 0.698 |
| ≥45 | 32 | 11 | 21 |  |
| T |  |  |  |  |
| T1/T2 | 46 | 17 | 29 | 0.395 |
| T3/T4 | 9 | 2 | 7 |  |
| N |  |  |  |  |
| Yes | 2 | 0 | 2 | 0.295 |
| No | 53 | 19 | 34 |  |
| M |  |  |  |  |
| Yes | 1 | 0 | 1 | 0.463 |
| No | 54 | 19 | 35 |  |
| STAGE |  |  |  |  |
| Ⅰ/Ⅱ | 48 | 17 | 31 | 0.722 |
| Ⅲ/Ⅳ | 7 | 2 | 5 |  |
| Capsular invasion |  |  |  |  |
| Yes | 56 | 19 | 37 | 0.315 |
| No | 2 | 0 | 2 |  |
| Vascular invasion |  |  |  |  |
| Yes | 23 | 6 | 17 | 0.380 |
| No | 35 | 13 | 22 |  |

**Supplementary Table S5 Sequence of primers used in this study.**

| Primer Name | Sequence (5’-3’) |
| --- | --- |
| CCL15-F | GCTAGCAGGAGGATGAAGGTCTCCGTGG |
| CCL15-R | CTCGAGTTATATTGAGTAGGGCTTCAGC |
| CCR1-F | AGCTGTCCGTTTGATTTTTGTCA |
| CCR1-R | CCAGGTCCAAATGTCTGCTCT |
| PDGF-BB-F | CTCGATCCGCTCCTTTGATGA |
| PDGF-BB-R | CGTTGGTGCGGTCTATGAG |
| IGF1-F | GCTCTTCAGTTCGTGTGTGGA |
| IGF1-R | GCCTCCTTAGATCACAGCTCC |
| IGFBP2-F | GACAATGGCGATGACCACTCA |
| IGFBP2-R | CAGCTCCTTCATACCCGACTT |
| CSF1-F | GGAGACCTCGTGCCAAATTA |
| CSF1-R | TATCTCTGAAGCGCATGGTG |
| CCL2-F | CTGTCTCAGCCAGATGCAGTT |
| CCL2-R | GAGCTTGGTGACAAATACTACA |
| CCL7-F | CTCAGCCAGTTGGGATTAATAC |
| CCL7-R | CACAGATCTCCTTGTCCAGTTTG |
| ACTB-F | GTGGAATCTGCCAGGAGGTA |
| ACTB-R | TGACCACTTCTCTGCCTGC |
