# Supplementary figures and images for: Follicular thyroid carcinoma but not adenoma recruits tumor-associated macrophages by releasing CCL15
Source: BMC Cancer. 2016 Feb 15;16:98. doi: 10.1186/s12885-016-2114-7 (PMC4753660; doi:10.1186/s12885-016-2114-7)

# Supplemental Figure S1

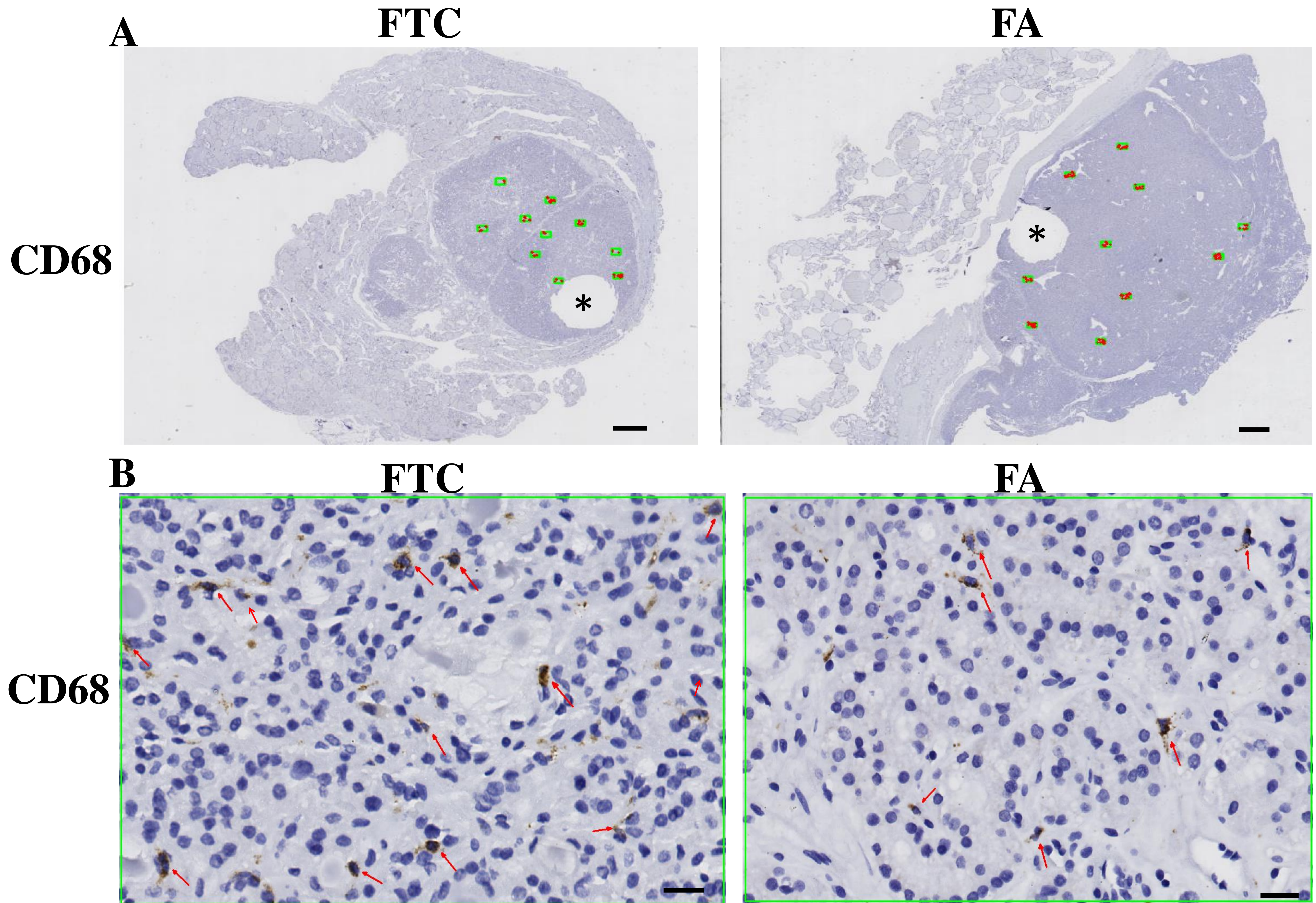

Supplemental Figure S2

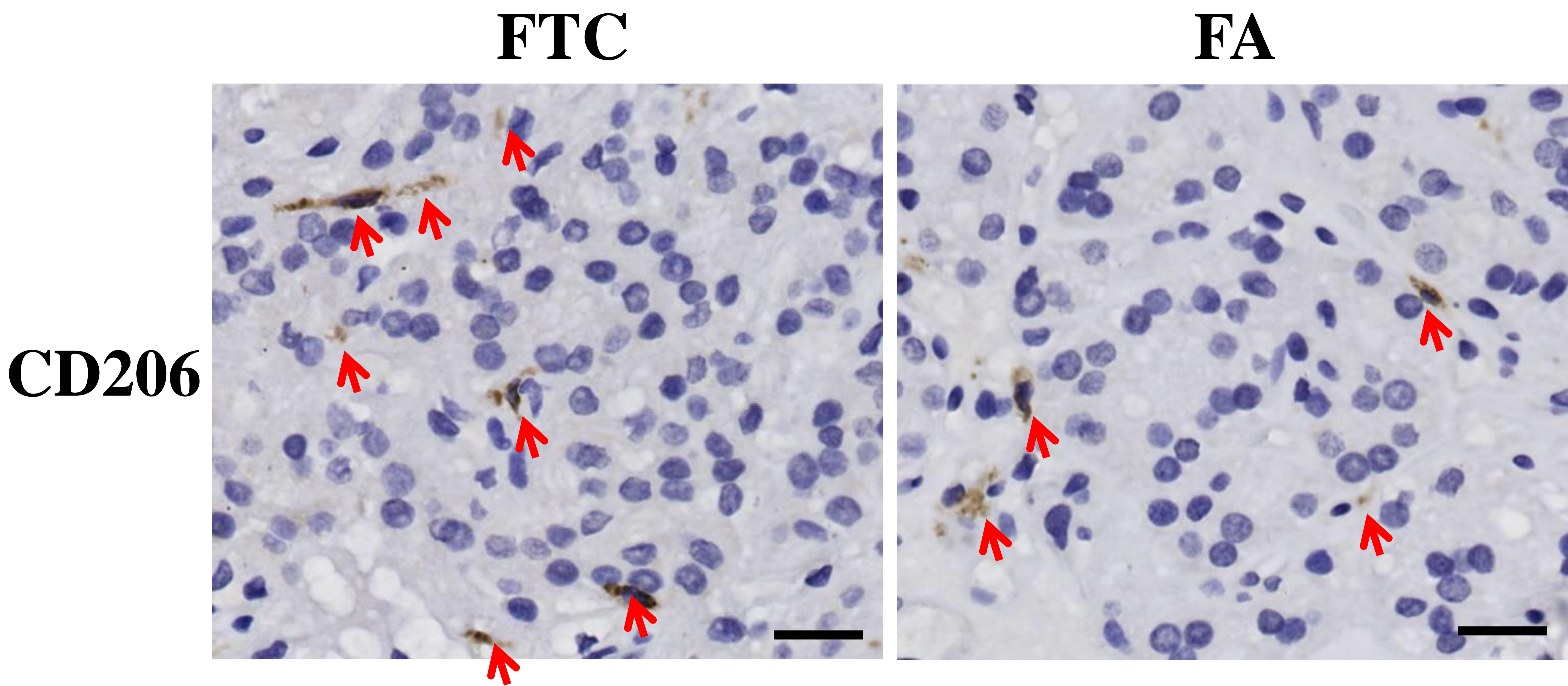

Supplement: Additional file 2: Figure S1 and S2. — Immunostain of CD68 in entire tissue of FTC/FA samples. (A) An example of immunohistochemistry analysis of CD68 in whole tissue samples of FTC (left panel) and FA (right panel). Stars indicated blank areas were taked out for tissue microarrays constructing. CD68+ cells in ten 200 μm *300 μm areas (green-red marked) of every sample were counted. Bar = 1 mm. (B) Enlarged picture of one count area (one green-red area in A). Arrows indicate the CD68+ macrophages in FTC (left panel) or FA (right panel). Bar = 20 μm. Figure S2. Densities of CD206+ cells in FTC are significantly higher than those in FA. Immunohistochemistry analysis of CD206 in 55 cases of tissue samples from FTC and FA patients. Arrows indicate the CD206+ macrophages. Bar = 20 μm. (PDF 509 kb) [file 12885_2016_2114_MOESM2_ESM.pdf]
